# Effects of deep sedation or general anesthesia on cardiac function in mice undergoing cardiovascular magnetic resonance

**DOI:** 10.1186/1532-429X-11-16

**Published:** 2009-05-19

**Authors:** Christopher J Berry, Daniel R Thedens, KellyAnn Light-McGroary, Jordan D Miller, William Kutschke, Kathy A Zimmerman, Robert M Weiss

**Affiliations:** 1 Division of Cardiovascular Medicine, Roy J. and Lucille A. Carver College of Medicine, University of Iowa, Iowa, USA; 2 Department of Radiology, Roy J. and Lucille A. Carver College of Medicine, University of Iowa, Iowa, USA; 3 Iowa City Veterans Affairs Medical Center, Iowa City, Iowa USA

## Abstract

**Background:**

Genetically engineered mouse models of human cardiovascular disease provide an opportunity to understand critical pathophysiological mechanisms. Cardiovascular magnetic resonance (CMR) provides precise reproducible assessment of cardiac structure and function, but, in contrast to echocardiography, requires that the animal be immobilized during image acquisition. General anesthetic regimens yield satisfactory images, but have the potential to significantly perturb cardiac function. The purpose of this study was to assess the effects of general anesthesia and a new deep sedation regimen, respectively, on cardiac function in mice as determined by CMR, and to compare them to results obtained in mildly sedated conscious mice by echocardiography.

**Results:**

In 6 mildly sedated normal conscious mice assessed by echo, heart rate was 615 ± 25 min^-1 ^(mean ± SE) and left ventricular ejection fraction (LVEF) was 0.94 ± 0.01. In the CMR studies of normal mice, heart rate was slightly lower during deep sedation with morphine/midazolam (583 ± 30 min^-1^), but the difference was not statistically significant. General anesthesia with 1% inhaled isoflurane significantly depressed heart rate (468 ± 7 min^-1^, p < 0.05 vs. conscious sedation). In 6 additional mice with ischemic LV failure, trends in heart rate were similar, but not statistically significant. In normal mice, deep sedation depressed LVEF (0.79 ± 0.04, p < 0.05 compared to light sedation), but to a significantly lesser extent than general anesthesia (0.60 ± 0.04, p < 0.05 vs. deep sedation).

In mice with ischemic LV failure, ejection fraction measurements were comparable when performed during light sedation, deep sedation, and general anesthesia, respectively. Contrast-to-noise ratios were similar during deep sedation and during general anesthesia, indicating comparable image quality. Left ventricular mass measurements made by CMR during deep sedation were nearly identical to those made during general anesthesia (r^2 ^= 0.99, mean absolute difference < 4%), indicating equivalent quantitative accuracy obtained with the two methods. The imaging procedures were well-tolerated in all mice.

**Conclusion:**

In mice with normal cardiac function, CMR during deep sedation causes significantly less depression of heart rate and ejection fraction than imaging during general anesthesia with isoflurane. In mice with heart failure, the sedation/anesthesia regimen had no clear impact on cardiac function. Deep sedation and general anesthesia produced CMR with comparable image quality and quantitative accuracy.

## Background

Genetically engineered mice are useful for mechanistic studies of cardiovascular disease states, which depend, in part, on quantitative phenotypic characterization. Cardiovascular magnetic resonance (CMR) provides quantitatively precise characterization of cardiac structure and function noninvasively *in vivo*, but can be somewhat hindered by the need to immobilize the animal during image acquisition. This is often accomplished by administration of general anesthetic agents, all of which are known to perturb cardiac function.[1,2] Echocardiography can be performed in unsedated or minimally sedated conscious mice, avoiding anesthesia-related functional artifacts, but only provides images in a limited number of planes and is quantitatively less accurate than CMR, especially in eccentrically remodeled ventricles.[3]

Here we introduce a new regimen of deep sedation with morphine and midazolam for mice undergoing CMR, and compare its effects on cardiac function to those observed during general anesthesia with isoflurane, using echocardiographic methods during mild conscious sedation as a reference standard for a "physiological preparation". We report that deep sedation caused no statistically significant depression of heart rate in normal mice, compared to conscious sedation, produced significantly less depression of systolic function, and yielded comparable image quality, compared to general anesthesia with isoflurane. In mice with heart failure, there was a non-significant trend toward preservation of physiologic heart rate during deep sedation, but no discernable differential effect on LV systolic function.

## Methods

All procedures were approved by the Office of Animal Resources at the University of Iowa.

### Mice

A group of 6 mice (3 male, age 6 ± 1 month) without known cardiovascular abnormalities, bred on a C57Bl/6 background and heterozygous for a null allele of interleukin-10, were identified from a breeding colony and entered this study as "normal" mice". An additional 6 mice (4 male, age 5 ± 2 months), heterozygous for deficiency of copper-zinc superoxide dismutase underwent surgical ligation of the proximal left coronary artery (see below), and entered the study as a group with "ischemic left ventricular failure".

### Surgical Preparation

Mice in the ischemic left ventricular failure group were anesthetized with ketamine/xylazine (87.5/12.5 mg/kg, respectively), and the anterior descending branch of the left coronary artery was ligated near the left atrial appendage, using 8-0 Ethilon suture (Ethicon). Successful ligation of the artery was confirmed by blanching of the myocardium. All wounds were closed with 6-0 silk, and animals were allowed to recover for 3 weeks.

### Echocardiography

Echocardiography was performed as previously described [4]. Briefly, mice were minimally sedated with midazolam, 0.15 mg by subcutaneous injection. This produced a mouse that was conscious, mildly curious, but docile. The anterior chest was shaved and warmed gel was applied, to improve acoustic interface. The mouse was cradled in the investigator's left hand and images were obtained in real-time using a 15 MHz linear-array transducer, coupled to a Sonos 5500^R ^ultrasonograph (Philips, Bothell, WA USA). Two-dimensional images were acquired at ~200 sec^-1 ^in parasternal long- and short-axis planes, and stored off-line for subsequent analysis. Pulse-wave Doppler interrogation of mitral inflow was used to measure heart rate.

### Echocardiography image analysis

Echocardiography image analysis was performed as previously described and validated.[5] Briefly, endo- and epicardial borders were traced electronically at end-diastole and end-systole using custom software developed for this purpose (Freeland Medical Systems, Indianapolis, IN). Left ventricular end-diastolic volume, end-systolic volume, stroke volume, ejection fraction, and mass were calculated using the bi-plane area-length method, previously validated in our laboratory.

### Preparation for CMR

Deep sedation was achieved by subcutaneous injection of morphine, 4.5 mg/kg, and midazolam, 9 mg/kg. After confirmation of loss of response to noxious stimuli, cutaneous CMR-compatible ECG leads and a rectal temperature probe were applied, the mouse was wrapped lightly in gauze, and inserted head-first into an imaging cone. Core temperature was monitored with a rectal probe, and supplemented with an external forced-air heat source so as to maintain core temperature ≥ 35°C. In 3 mice with ischemic heart failure, probe insertion was not possible due to perineal edema or other factors. In those cases, forced air settings were selected to coincide with those used in mice with temperature probe in place.

During a separate imaging session, general anesthesia was induced by inhalation of 3% isoflurane for 3 minutes, followed by steady-state inhalation of 1% isoflurane and room air via nose cone. The remainder of animal preparation was the same as for deep sedation.

### CMR

CMR was performed using a Unity/Inova 4.7T horizontal bore scanner (Varian, Palo Alto, CA) equipped with a small animal receiver coil (Doty Scientific, Columbia, South Carolina, USA). After acquisition of axial and oblique localizers, image data were acquired using an ECG-gated short-axis multi-slice cine spoiled gradient echo pulse sequence covering the entire LV throughout the cardiac cycle with parameters TR/TE = 6.0/3.4 ms, flip angle = 15°, using 1 mm slices with a 256 × 128 pixel matrix covering a 56 mm × 28 mm field of view in each slice, yielding 15–20 cine frames per cardiac cycle depending on the heart rate. Imaging for the purpose of ejection fraction determination commenced within 10 minutes after confirmation of sedation or anesthesia. Heart rates were measured simultaneously with image acquisition. Respiratory monitoring was not performed. Images were batched and archived off-line for later analysis.

### CMR image analysis

Images were viewed in cine mode to confirm completeness of the data set, spanning at least one slice below the left ventricular apex, and one slice above the aortic valve, and for a complete cardiac cycle. End-diastolic and end-systolic frames were identified, and endocardial and epicardial borders were planimetered electronically using standard software (Medis^R^, Rotterdam). Cardiac volumes and mass were calculated using modified Simpson's Rule, and tabulated for each mouse under each experimental condition.

In order to objectively evaluate the effect of sedation vs. general anesthesia on image quality, contrast-to-noise ratio (CNR) was measured in two contiguous mid-ventricular slices from each mouse under both experimental conditions, using the following formula:



where S.I. = signal intensity, myo = anterior left ventricular myocardium, and S.D. = standard deviation.

### Experimental protocol

On Day 1, 6 mice underwent surgical ligation of the left coronary artery; 6 additional normal mice had no surgical procedure. On Day 22 of the study, each mouse underwent echocardiography during mild conscious sedation. On Day 25, each mouse underwent CMR during deep sedation. On Day 40 ± 7, each mouse underwent CMR during general anesthesia. Because some of the mice were participants in long-term molecular and genetic studies, none were euthanized for the purpose of postmortem validation of image findings.

### Statistics

Data are reported as mean ± SE. Group data obtained during conscious sedation, deep sedation, and general anesthesia, respectively, were compared using paired ANOVA. Results were considered statistically significant if p ≤ 0.05.

## Results

All 12 mice completed each of 3 imaging procedures, and resumed normal in-cage activity thereafter.

### Image Quality

**Figure 1:**
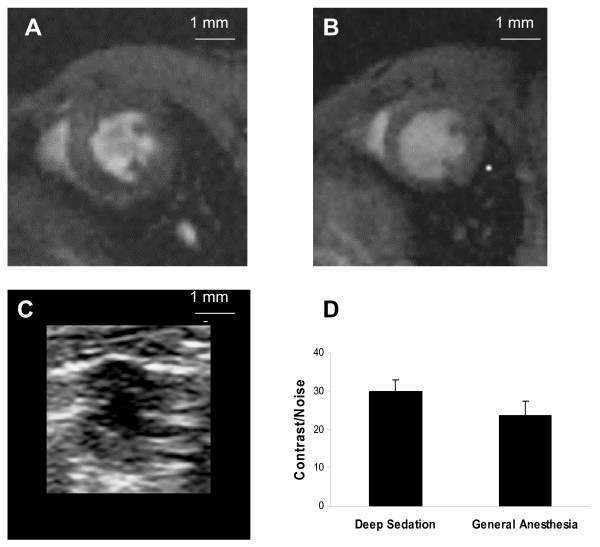
**Image Quality**. Mid-ventricular short-axis end-diastolic images acquired (A) with CMR during deep sedation, (B) with CMR during general anesthesia, and (C) with echocardiography, all from the same mouse. Images are shown to scale, white bar = 1 mm. (D) Comparison of contrast-to-noise ratio for CMR obtained during deep sedation vs. general anesthesia, p = 0.17.

Figure 1 shows representative short-axis end-diastolic images from a single mouse, acquired by each of the 3 imaging methods. Panel D shows group-averaged contrast-to-noise ratios for CMR images during deep sedation and general anesthesia, respectively. The two CMR techniques provided similar levels of image quality.

### Effects of sedation or anesthesia on heart rate

During mild conscious sedation, normal mice had a heart rate of 615 ± 25 min^-1^. The effects of deep sedation and general anesthesia, respectively, are shown in the Table. Deep sedation caused approximately 5% decrease in heart rate (p = NS). However, general anesthesia caused a highly significant 24% drop in heart rate, compared to mild conscious sedation. In mice with heart failure, there was a statistically non-significant trend toward lower heart rate during general anesthesia, compared to either light or deep sedation, respectively.

### Effects of sedation or anesthesia on left ventricular ejection fraction (Table 1)

**Table 1:** Image-derived LV parameters.

	**Normal mice (N = 6)**		
	Conscious sedation	Deep sedation	General Anesthesia

	Echocardiography	MRI	MRI

Heart rate (min^-1^)	615 ± 25	583 ± 30	468 ± 17*,**
LVEF	0.94 ± 0.01	0.79 ± 0.04*	0.60 ± 0.04*,**
LV mass (mg)	N/A	67 ± 7	66 ± 6
Temperature (°C)	N/A	35.5 ± 0.6	35.4 ± 0.6
CNR	N/A	36 ± 3	32 ± 7

	**Heart failure (N = 6)**		

	Conscious sedation	Deep sedation	General Anesthesia

	Echocardiography	MRI	MRI

Heart rate (min^-1^)	508 ± 32	536 ± 38	416 ± 44
LVEF	0.24 ± 0.06	0.20 ± 0.04	0.19 ± 0.05
LV mass (mg)	N/A	124 ± 18	126 ± 18
CNR	N/A	26 ± 7	18 ± 4

*p < 0.05 vs. conscious sedation

**p < 0.05 vs. deep sedation

During mild conscious sedation, normal mice had LVEF = 0.94 ± 0.01. Deep sedation caused a statistically significant decrease in LVEF of 16%, compared to mild conscious sedation. However, general anesthesia caused a more profound 36% decrease in LVEF (p < 0.05 vs. deep sedation). In mice with ischemic left ventricular dysfunction, ejection fractions were proportionally more variable than in normal mice. When analyzed separately, heart failure mice demonstrated no discernable effect of sedation vs. anesthesia upon the measured ejection fraction.

**Figure 2:**
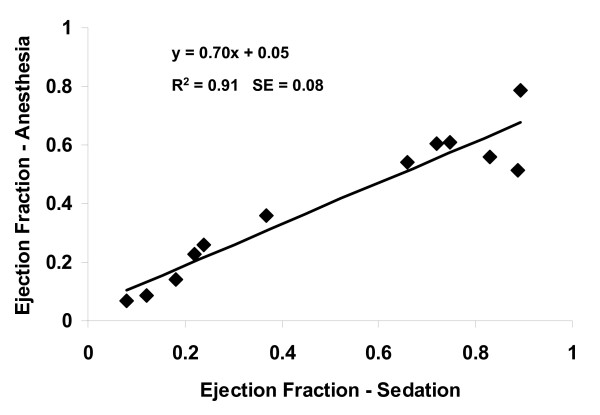
**Ejection Fraction**. General anesthesia caused a systematic 30% decrease in left ventricular ejection fraction, compared to measurements made during deep sedation. The relationship was relatively consistent over the whole range represented by normal (n = 6) and LV failure (n = 6) mice.

Comparison of ejection fractions obtained during deep sedation to those obtained during general anesthesia is shown graphically in Figure 2. The data indicate a systematic trend toward lower LVEF during general anesthesia, when all mice are considered.

### Effect of deep sedation or anesthesia on core temperature

In normal mice, there was no difference in core body temperature during deep sedation vs. general anesthesia (Table). Furthermore, the setting on the forced air heat source required to maintain this temperature was the same for both conditions. In mice with ischemic heart failure, insertion of the temperature probe was impossible or poorly tolerated in 3 mice, due to perineal edema or other factors. For those animals, forced air settings were empirically set to match those for normal mice (data not shown).

### Effect of deep sedation or anesthesia on quantitative accuracy

**Figure 3:**
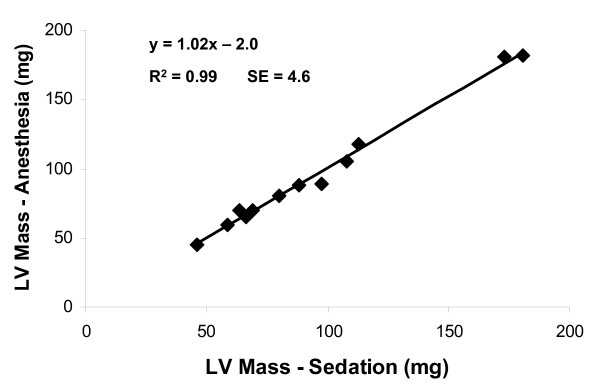
**Left ventricular mass**. Mass measurements made during deep sedation and general anesthesia, respectively, were nearly identical across the entire range.

As noted, contrast-to-noise ratio was similar during imaging during deep sedation vs. imaging during general anesthesia. Whereas the sedation and anesthetic regimens caused disparate effects on heart rate and cardiac function, left ventricular mass should have remained nearly constant during the brief interval between studies in mature mice. Figure 3 shows the correlation between mass measurements made during each sedation or anesthesia regimen. Indeed the measurements are nearly identical over a broad range of LV masses.

## Discussion

The most important finding of this study is that it introduces a new deep sedation regimen for CMR of genetically engineered mice, which causes insignificant reduction in heart rate and only mild reduction in left ventricular systolic function in normal mice and in mice with ischemic left ventricular failure.

For these determinations, the reference standard was echocardiography performed during mild conscious sedation. The reported heart rate, 615 ± 25 min^-1^, and left ventricular ejection fraction, 0.94 ± 0.01, in normal mice, are nearly identical to previous studies performed on unsedated mice in our laboratory.[6] Whereas all general anesthetic preparations are known to depress cardiac function [1,2], deep sedation with morphine and midazolam produces no significant decrement in heart rate, and produces very significant improvement in left ventricular systolic function, when compared to general anesthesia with isoflurane. These attributes accrue without sacrificing image quality or the quantitative accuracy of CMR studies, despite higher heart rates during deep sedation.

Isoflurane itself has become a preferred general anesthetic agent for cardiovascular phenotyping studies, because it causes less depression of cardiac function than injectable general anesthetics.[2] In the present study, LVEF measurements made during general anesthesia, while significantly depressed, maintained a consistent linear relationship to measurements made during deep sedation. Thus, carefully controlled studies using isoflurane continue to report mechanistically valid results in genetically engineered models of cardiovascular disease in mice [7-14].

Clinically, however, patients with decompensated heart failure or severe valvular heart disease are considered at "high risk" for procedures requiring general anesthesia. Similar concerns can arise in the study of mice with, for example, severe aortic stenosis [4]. Thus, concerns about the risks of general anesthesia in potentially valuable mice may limit the ability to assess cardiovascular function with methods such as CMR. Our finding that deep sedation results in less depression of heart rate and systolic function may broaden the applicability of CMR methods in "sick" mice.

In the present study, we report LVEF for normal mice during isoflurane = 0.60, which is consistent with a number of previous reports [8,14], but significantly lower than others [7]. Yang *et al *employed high-dose diazepam for sedation during CMR, and reported normal mean heart rate and ejection fraction of 494 and 0.65, respectively [15], which are similar to reported values for isoflurane, but significantly lower than results reported here during deep sedation with morphine/midazolam. It cannot be determined whether these differences related to differences in the respective groups of "normal" mice, as opposed to differential effects of the sedation regime. A feature of the present study is that it compares cardiac function in the same mice, subjected to 3 different sedation/anesthesia regimens.

This study has some limitations. It was not possible to measure core temperature during echocardiography in conscious mice. Because the mice remained capable of ambulation and maintained normal heart rates, it could be inferred that core temperature was normal, i.e. higher than during CMR. It is possible that temperature differences accounted for some of the observed effects on cardiac function. However, it is noted that there was no significant difference in heart rates during deep vs. light sedation. Furthermore, temperatures during deep sedation were similar to temperatures during general anesthesia, whereas cardiac effects were significantly different. Taken together, these findings argue for differential direct effects of the sedation/anesthesia agents, independent of their effects on core temperature.

Paired CMR studies were performed 15 days apart, after healed myocardial infarction, during which cardiac function may have changed. The mice strains selected for these studies were from only two genotypes – heterozygous for deletion of IL-10 ("normal mice") or copper-zinc superoxide dismutase (heart failure mice). While those heterozygous mice are not known to exhibit impaired cardiac function at the ages studied, it is possible that there were genotype-specific responses to sedation or anesthesia that would not extrapolate to other strains.

The superior safety profile of the deep sedation regimen was not specifically addressed. Indeed, all mice survived the entire experimental protocol, including imaging during general anesthesia. However, the perception of increased risk of general anesthesia in sick mice is widely held, and this continues to limit the access of those mice to CMR at our center, and others.

The imaging protocols employed for these studies were relatively simple, requiring less than 20 minutes. More complex protocols might outlast the effects of a single subcutaneous injection of morphine/midazolam, whereas inhalation of isoflurane would be expected to yield a consistent effect over time. This consideration is offset by the simplicity of the deep sedation regimen, which avoids the necessity for ambient air-handling procedures and other safeguards necessary for handling volatile agents.

*A priori*, it is difficult to determine "normal" cardiovascular function in mice. We have previously observed that mild conscious sedation produces no significant perturbation of heart rate or systolic function in mice, compared to the unsedated state, and we report relatively narrow ranges of normal.[4,6] However, confrontation by investigators may introduce artifactual cardiostimulatory or depressant influences which are difficult or impossible to account for. It will therefore remain important to include carefully configured "control" groups in all mechanistic studies.

## Conclusion

Deep anesthesia with morphine and midazolam causes less cardiac depression during magnetic imaging studies than general anesthesia with isoflurane, without sacrificing image quality. The new method of preparing genetically engineered mice for quantitative cardiovascular assessment may broaden applications for CMR in this rapidly growing and evolving field.

## Abbreviations

LV: left ventricle; EF: ejection fraction; CMR: cardiovascular magnetic resonance; CNR: contrast-to-noise ratio

## Competing interests

The authors declare that they have no competing interests.

## Authors' contributions

CB was involved in experimental design, CMR data acquisition, and manuscript writing. DT designed CMR image-acquisition and image-quality assessment methods. KL-M acquired and analyzed some echo and CMR images. JM participated in echocardiographic image acquisition. WK performed animal surgeries and participated in drafting and critically reviewing the manuscript. KZ analyzed and tabulated echocardiographic image data. RW designed the study and analyzed echocardiographic and CMR data, and critically revised the manuscript.
